# Is static or cidal antibiotic falsifiable?

**DOI:** 10.1128/aac.01361-25

**Published:** 2025-10-13

**Authors:** Santanu Datta

**Affiliations:** 1 Bugworks Research, C-CAMP, GKVK Campus 668176 , Bangalore, India; Columbia University Irving Medical Center, New York, New York, USA

**Keywords:** cidal, static, antibiotic

## LETTER

I read with great interest the commentary by Spellberg et al., *“Static vs. cidal: it’s not complex; it’s simply incorrect”* (1). While I agree with the authors that the conventional bacteriostatic versus bactericidal dichotomy is of limited value for guiding antibiotic therapy, I respectfully caution against the absolutist framing that there are *no differences* between these classes of agents in humans.

Karl Popper, while outlining the philosophy of science, emphasized that the hallmark of a scientific hypothesis is its falsifiability. Scientific claims should remain provisional, open to refutation by future evidence. Declaring the static–cidal concept “simply incorrect” risks stepping outside this principle, transforming an empirical matter into an unfalsifiable statement of doctrine.

*In vitro*, antibiotic activity is largely monoparametric—the outcome mainly reflects the action of the compound under specified conditions. Under such conditions, distinctions between static and cidal activity can be observed, whether in MBC/MIC ratios or in phenomena such as reactive oxygen species generation associated with certain cidal drugs. These differences are tangible, measurable, and therefore falsifiable.

In contrast, *in vivo* infection is multiparametric: antibiotic exposure, pathogen physiology, host immunity, tissue penetration, inoculum effect, and comorbid conditions all interact. This complexity can obscure mechanistic distinctions and explain why randomized clinical trials to date often fail to show consistent outcome differences between static and cidal regimens. However, this should not be regarded as proof that no differences exist. Specific contexts—such as profound immunosuppression, infections in sanctuary sites, or high-inoculum states—may still uncover clinically relevant distinctions, and these remain valid areas for further investigation.

A more balanced conclusion would be that current evidence shows no consistent superiority of cidal over static agents in most clinical settings, while recognizing that mechanistic differences observed *in vitro* retain explanatory value and may be relevant under specific host or infection conditions. Such framing aligns more closely with Popper’s principle: supported by data, but always open to challenge by future experiments.
